# Primary health care organizational characteristics associated with better accessibility: data from the QUALICO-PC survey in Quebec

**DOI:** 10.1186/s12875-018-0871-x

**Published:** 2018-12-03

**Authors:** Andrée-Anne Paré-Plante, Antoine Boivin, Djamal Berbiche, Mylaine Breton, Maryse Guay

**Affiliations:** 1 Département de Médecine de Famille et de Médecine d’Urgence de l‘Université de Sherbrooke, Campus de Longueuil, 150 place Charles-Le Moyne, 9e étage, Longueuil, J4K 0A8 Canada; 2 Centre de recherche Charles-Le Moyne - Saguenay–Lac-Saint-Jean sur les innovations en santé, Campus de Longueuil, 150 place Charles-Le Moyne, 9e étage, Longueuil, J4K 0A8 Canada; 3 Département de Médecine de Famille et Médecine d’Urgence de l’Université de Montreal, Montreal, Canada; 4 Centre de Recherche du Centre Hospitalier de l’Université de Montréal, Montreal, Canada; 5 Institut de Recherche en Santé Publique de l’Université de Montréal, Montreal, Canada; 6 Département des Sciences de la Santé Communautaire de l’Université de Sherbrooke, Sherbrooke, Canada; 7 Direction de Santé Publique, CISSS de la Montérégie-Centre, Longueuil, Canada

**Keywords:** Primary health care, Quebec, Survey, Secondary data analysis, First-contact accessibility

## Abstract

**Background:**

First-contact accessibility remains an important problem in Canada, with this indicator staying the worst of all Organization for Economic Co-operation and Development countries. In the province of Quebec, a number of primary healthcare (PHC) organizations have adopted measures to improve access (e.g. advance access scheduling, expanded nursing role, electronic medical record, financial incentives). The impact of those changes is unknown. The goal of this study is to assess which PHC organizations’ characteristics are associated with improved first-contact accessibility.

**Methods:**

We conducted a secondary data analysis of the Quebec survey, conducted as part of the QUALICO-PC study on primary care performance. QUALICO-PC is a cross-sectional study to assess quality, costs and equity in PHC across 35 countries and jurisdictions. Organizational characteristics were measured from the family practitioners’ questionnaire. First-contact accessibility was measured from the patient questionnaire filled by patients who received care in the participating PHC organizations. Multi-level logistic regression was used to assess the association of organizational characteristics as predictors of patient-reported accessibility.

**Results:**

A total of 218 family practitioners participated in the study with 1798 of their patients. PHC organizations characteristics associated with increased first-contact accessibility included the possibility to have a same-day appointment or to walk in the clinic without an appointment, higher number of physicians per clinic and higher number of hours worked by the family physician. Electronic medical record and expanded nursing role were not associated with increased accessibility.

**Conclusions:**

Same-day access and higher family physician working hours are associated with improved patient-reported accessibility. Other PHC organizations characteristics targeted by recent reforms were not associated with improved accessibility.

## Background

Primary health care (PHC) is the first level of care for the patient, his family and his community as defined by WHO [1]. PHC serves four main functions: accessibility, continuity, comprehensiveness and coordination [2]. Accessibility is a dimension of the patient’s experience of care that can be measured to assess the performance of PHC. One of the indicators frequently used to measure accessibility is first-contact accessibility, defined by the ease to have a timely appointment with a PHC provider [3].

Different levels of PHC characteristics influence its performance [4]. The system level is where the government policies, the funding of health care and the provider remuneration act as factors on PHC. The context level is where the practice setting and its community integration affect PHC performance. Finally, the practice level is where specific characteristics of clinics influence their performance on care delivery. This study will look at organizational characteristics of PHC organizations, specifically resources and types and scope of services, and their effect on first-contact accessibility.

First-contact accessibility remains an important concern in Canada, with this indicator staying among the worst of all countries participating in international surveys on PHC performance [5, 6]. During the past decade, primary care reforms in the Canadian province of Quebec have sought to improve access to PHC [7, 8] and other dimensions of PHC performance [9]. It started with the implementation of Family Medicine Groups in 2002 and integration of the interdisciplinary team into these Family Medicine Groups in 2013 [10] with expanded nursing roles (nurse practitioners and clinical nurses in chronic disease prevention and management). Family Medicine Groups are family practitioners working in a clinic with other health professionals to dispense health services to a population of registered patients [11]. The electronic medical records were progressively used in a majority of organizations with incentives for PHC organizations to adopt them between 2002 and 2017 [10]. Since 2010, advanced access, a scheduling method designed to offer same-day appointments and meet the demand for care without a waiting-list, has been highly recommended through financial incentives and policies to be implemented across PHC organizations. All of these innovations were adopted with variation between practices. The actual impact of these changes in PHC practices on access, on which this article is focusing, is not well known.

In Quebec, prior to PHC reforms, attributes of PHC organizations associated with increased first-contact accessibility included the presence of a nurse and the number of family practitioners in the clinic being greater than 5 [9]. In a recent study conducted in Ontario (Canada), the number of years of practice of a family practitioner was the sole practice characteristic associated with increased first-contact accessibility [12]. However, the association between practice characteristics and first-contact accessibility has not been studied after PHC reforms in Quebec.

The **objective** of this study is to identify organizational characteristics of PHC practices associated with better first-contact accessibility.

## Methods

### Study design

This study is based on a secondary analysis of the Quebec survey data collected in a larger international cross-sectional study on PHC performance: the Quality and Costs of Primary Care in Europe (QUALICO-PC) Study [13]. QUALICO-PC is a cross-sectional survey started in 2010 to assess quality, costs and equity in PHC across 35 countries. Each participating Canadian province is being considered a separate jurisdiction in the QUALICO-PC design, with its own sampling but similar questionnaires [14]. The Quebec QUALICO-PC survey was conducted between 2013 and 2014. The complete methodology of QUALICO-PC study has been described elsewhere [13].

### Study population and sample

Two groups of participants were recruited: family practitioners in PHC organizations and patients receiving care within this PHC organization by the family practitioners. Family practitioners were identified through stratified random sampling of all Quebec family practitioners, with a maximum of one participating family physician per PHC organization. The sampling of the family practitioners was obtained from a random list of 2000 family practitioners stratified by four types of regions: 1) academic (having an established faculty of medicine with high density population), 2) peripheral (being close to the academic region with high density population), 3) intermediate (having regional health care centers with moderate density population), and 4) remote (being large territories with low density population). Inclusion criteria for the family practitioners were to provide PHC services and to have a valid e-mail address. The exclusion criteria were to practice in the isolated region of Northern Quebec, or to be disengaged from the public healthcare system (representing fewer than 3.2% of all family physicians in the province [15]).

Family practitioner participants were recruited by the QUALICO-PC study team in Quebec hosted at the Quebec National Institute of Public Health. They received two consecutive invitation e-mails to participate; if they had not responded, they received later a phone call by a member of the research team. Following the family practitioner’s recruitment, the research package was sent to his clinic: the family practitioner survey, the patient experience surveys as well as the consent forms for the participants.

Patients receiving usual care in participating PHC organizations were targeted for the experience of care survey. The inclusion criteria for the patients were to be 18 years of age and over, and to speak and understand written French or English. Patients were recruited by receptionists of participating PHC clinics. The first ten patients of the participating family practitioner who presented themselves on a typical day of work were asked to participate in the study. All participants provided written informed consent.

### Data source

Two types of surveys were used to collect in-depth information regarding primary care activities: providers and patients. The surveys were developed by the QUALICO-PC team and published elsewhere [16]. They were translated in French by the Quebec research team. The family practitioner survey provided data on PHC organizational characteristics. It was completed by family practitioners on paper and sent back by mail to the research team. The patient experience survey was completed on paper by the patients before and after their appointment with their family practitioner in the waiting room. The family practitioners did not know which of their patients participated. The patient experience survey were also sent back by mail to the research team.

### Conceptual model

**Fig. 1:**
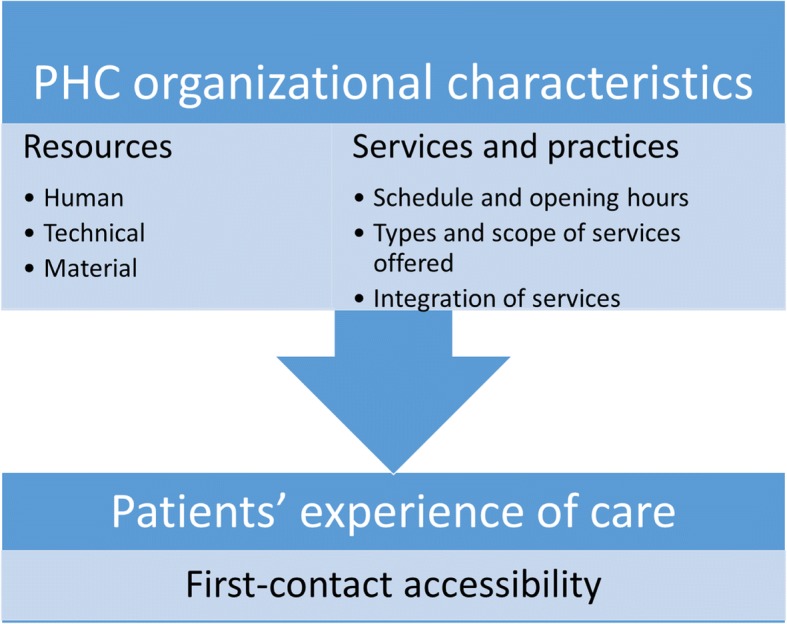
Conceptual model. Legend: Adapted from Hogg et al. [4] and Lévesque et al. [14]

The conceptual model used in this study is shown in Fig. 1. It was adapted from Hogg and al. [4], which postulates an association between PHC organizational characteristics and its performance, including first-contact accessibility. PHC organizational characteristics are further classified according to dimensions developed by Lévesque and al. [17].

### Study variables

The questions in the surveys were of different types: scaled questions, open-ended questions with numerical answers, multiple choice questions, and dichotomous questions. The independent variables are the PHC organizational characteristics (Fig. 1): number of family practitioners and physicians of all specialties in the clinic, use of specific equipment and/or electronic medical records, schedule and opening hours of the clinic, number of hours worked and the number of patients seen by the doctor on a typical day, and the presence of a nurse providing primary care services in the PHC organization. The dependent variable is first-contact accessibility, as reported by the patient participants when answering yes or no to the question: “Were you able to get the appointment with your doctor as quickly as you wanted?” This question was well validated [16, 18] and is considered to be the best question of the QUALICO-PC questionnaires to measure first-contact accessibility [19].

Participant characteristics were used as co-variates, including patients’ characteristics (sex, age, income, level of fluency in French or English, education, general health status and presence of chronic conditions) and family practitioners’ characteristics (sex and age).

### Analysis

Descriptive participant characteristics (patients and family practitioners) were assessed. All data were analysed in a multilevel model as the patient data was nested in the family practitioner’s data. The first step was identifying associations between PHC organizations’ characteristics and first-contact accessibility, using a bivariate regression analysis. Then, using a multivariate regression analysis, where all the variables were included, adjustment was made for possible confounding factors (patients’ characteristics and practitioners’ characteristics). Statistical significance was set at *p* < 0.05. All analyses were performed with Statistical Analysis System (SAS Institute Inc. Cary, NC, version 9.4).

## Results

In total, 218 family practitioners working in 194 different PHC organizations participated in the study with 1798 of their patients. The response rate for the family practitioners was 17% with an average of 8 patient participants each. The descriptive analysis of both samples of patients and family practitioners are presented in Table 1.

**Table 1:** Participant characteristics

	Frequency (n)	Proportion (%)	Khi-2 (*p*-value)
Patients			
Sex	1765^a^		0.1164
Male	593	33.6	
Female	1172	66.4	
Age	1752^a^		0.4720
18–25	100	5.7	
25–34	213	12.2	
35–44	249	14.2	
45–54	351	20.0	
55–64	393	22.4	
65 and over	446	25.5	
Income	1731^a^		0.4777
Below average	377	21.8	
About average	1011	58.4	
Above average	343	19.4	
Fluency in French or English	1752^a^		0.3931
Fluent	1520	86.8	
Sufficiently	140	8.0	
Moderately	46	2.6	
Little	35	2.0	
Not at all	11	0.6	
Education	1749^a^		0.2534
No qualifications, primary, or lower secondary	275	15.7	
Upper secondary education (grades 10–12)	622	35.6	
Post-secondary education (college, undergraduate)	852	48.7	
Employment status	1798^a^		0.0340
Working	960	53.4	
Retired	523	29.1	
Unable to work	142	7.9	
Other	192	10.7	
General health status	1778^a^		0.0044
Very good	416	23.4	
Good	881	49.6	
Fair	420	23.6	
Poor	61	3.4	
Chronic conditions	1759^a^		0.2394
Yes	918	52.2	
No	841	47.8	
Family practitioners			
Sex	215^a^		0.5886
Male	97	45.1	
Female	118	54.9	
Age	211^a^		0.6703
25–34	34	16.1	
35–44	31	14.7	
45–54	64	30.3	
55 and over	82	38.9	

^a^Total n are different due to different partial non-response rate to each question

The study sample for the family practitioners is representative for age and sex (FMOQ 2013). Females patients were over-represented (two thirds of respondents), but the patient sample was representative of the Quebec population for education and presence of a chronic illness [6].

In participating PHC organizations, the total number of family practitioners ranged from one to 31, the median being 4.75 family practitioners per clinic. The scope of services offered by family practitioners in the clinics varied between 3 and 80 h worked per week (median of 38.5) as a family practitioner and between 8 and 53 contacts with patients on a typical day (median of 27.5). The number of physicians from other disciplines in the clinic, ranged from none (85.6%) to between one and 25 (14.6%). The majority of clinics offered patients the possibility to have a same-day appointment or to walk in the clinic without an appointment (95.2%) and most patients (76.1%) were able to get an appointment with their doctor as quickly as they wanted.

Table 2 shows the association of PHC characteristics and access using non-adjusted and adjusted odds ratios. Using the bivariate (non-adjusted) model, the only PHC characteristic associated with increased first-contact accessibility was the possibility to have a same-day appointment or to walk in the clinic without an appointment. When adjusting for family practitioners’ and patients’ characteristics in the multilevel analysis model, two more independent variables were associated with increased first-contact accessibility: increased number of physicians from other disciplines in the clinic and increased number of hours worked per week by the family practitioner. Technical resources with the presence of specialized equipment in the clinic (mainly radiology and surgical equipment) was associated with decreased accessibility. The presence of nurses offering primary care services in the clinic was not associated with first-contact accessibility.

**Table 2:** Association of organizational characteristics with patient-reported first-contact accessibility

Organizational characteristics	OR	CI 95%	*p*-value	Adjusted OR^*^	CI 95%^*^	*p*-value^*^
Human resources						
Number of family practitioners in the clinic^a^	1.00	(0.97–1.03)	0.89	0.96	(0.89–1.03)	0.27
Number of physicians from other disciplines in the clinic^a^	1.05	(0.996–1.10)	0.07	1.13	(1.03–1.24)	0.01
Technical and material resources						
Presence of specific equipment in the clinic^c^	0.98	(0.95–1.00)	0.08	0.89	(0.83–0.96)	0.002
Use of information technology for different tasks in the clinic^d^	0.98	(0.94–1.02)	0.37	1.02	(0.92–1.14)	0.67
Schedule and opening hours						
Typical amount of time scheduled for a regular visit with the family practitioner (in minutes)^a^	1.00	(0.98–1.02)	0.89	1.03	(0.99–1.07)	0.21
Number of opening hours per day^a^	1.01	(0.97–1.05)	0.54	1.10	(0.96–1.26)	0.18
Percentage of visits with a scheduled appointment^a^	1.00	(0.999–1.01)	0.12	1.00	(0.98–1.01)	0.66
Possibility to obtain a same-day appointment or to walk-in the clinic without an appointment^b^	1.66	(1.01–2.74)	0.04	2.94	(1.15–7.51)	0.02
Type and scope of services						
Number of hours worked per week by the family practitioner^a^	1.00	(0.99–1.01)	0.39	1.03	(1.00–1.06)	0.03
Number of hours per week worked by the family practitioner in this clinic^a^	1.00	(0.99–1.01)	0.56	0.99	(0.97–1.01)	0.26
Number of contacts with patients by the family practitioner in a typical day^a^	0.99	(0.98–1.00)	0.30	0.99	(0.97–1.01)	0.42
Integration of services						
Primary care services offered independently by a nurse in the clinic^e^	1.03	(0.90–1.18)	0.69	1.03	(0.90–1.18)	0.69

^*^Results are adjusted for patients and family practitioners characteristics

OR superior to 1 indicates an increase in first-contact accessibility

^a^Difference with every increase of one unit for these continuous variables (number of family practitioners, physicians, minutes)

^b^Difference between the presence or the absence of this characteristic

^c^Difference with every additional equipment present in the clinic

^d^Difference with every additional task performed with information technology

^e^Difference with every additional service provided independently by a nurse

## Discussion

Accessibility, especially first-contact accessibility is a fundamental issue in contemporary PHC. To our knowledge, this is the first study to examine the association between practice characteristics and better first-contact accessibility after PHC reforms in Quebec. The main finding of this study was that first-contact accessibility increased almost threefold when there was a possibility for patients to get a same-day appointment or walk-in clinic appointment in their own primary care practice. Practices where family practitioners worked longer hours per week also reported increased access.

Authors could not observe differences in access with two important targets of the PHC reforms: the use of electronic medical record and the presence of a nurse. Absence of association with nurses’ role is consistent with the findings of another study in Canada [12] but different from a study conducted before the PHC reform and Family medicine group implementation in Quebec [9]. Nurses’ roles in Quebec PHC during the past decade has focused mainly on increased uptake of preventive care and chronic disease management. It also has been a target of the reform to implement the goals of the patient-centered medical home into Family medicine groups [20]. The patient-centered medical home is an interprofessional model of PHC organization that aims at providing comprehensive care with particular objectives for quality, safety and access to care [20]. Accordingly, the effects of nurses in this context may have affected other dimensions of PHC performance (e.g. comprehensiveness and continuity) despite explicit policy objectives assuming that the presence of nurses would improve access. It is also possible that PHC organizations already working with nurses before the reform were highly performing clinics more opened to other types of innovations (including advanced access scheduling). In a recent article using QUALICO-PC data from all Canadian provinces, individual PHC practices were categorized as a traditional clinic model (eg. solo practitioners) or a new clinic model (eg. group practices). There were no differences in terms of accessibility between the models [21]. This is concordant with the observation that interprofessional PHC organizations tend to affect dimensions of the patient experience other than accessibility.

The number of physicians from other specialties working in the clinic was associated with better perceived accessibility by patients. However, this variable was not distributed normally and the majority of PHC organizations had no specialists. It is possible that well performing PHC organizations offered medical services not just from family practitioners or that presence of physicians from other disciplines in the clinic was associated with other characteristics of practices influencing access that we did not measure. It is concordant with findings from the Miedema et al. study mentioned before.

A surprizing result of this study is the overall high degree of perceived access by PHC patients, with over 76% of respondents being able to get an appointment as quickly as they want. Before the reform, only 10% of patients having access to a primary care provider in Quebec were confident they could get an appointment with their regular doctor if they urgently needed it [22]. This observed difference is important because in recent years, incentives for family practitioners in Quebec were to improve the ease to get a timely appointment with a family practitioner or with another healthcare professional. One should keep in mind that QUALICO-PC participants were recruited from patients who successfully obtained an appointment in primary care with their regular family practitioner, which may have overestimated our measures of patient-reported accessibility. Reported first-contact accessibility was much higher in our study than in general population surveys [6], but similar to studies surveying patients who already had access to a family practitioner [12].

### Limitations

The cross-sectional nature of the family practitioner survey and the patient experience survey could not allow us to establish causality between first-contact accessibility and organizational characteristics.

Other limitations are those related to a secondary data analysis study. The surveys were not conceptualized only to measure first-contact accessibility but all dimensions of PHC and they were created in Europe [16] although validated for use in Quebec [23].

A desirability bias could also have occurred for instance if practitioners tried to give a better overall picture of their practice or if patients would embellish their ease to get an appointment. This bias could therefore have the effect of incorrectly classifying responders. We have no way to evaluate the magnitude of this effect if it is present. Work records of the clinics were not available to confirm the data collected through the family practitioners’ answers.

The main limitation of this study is the low response rate of family practitioners (17%). Response rate in studies involving physicians is a common problem. The Quebec survey had the second highest response rate among Canadian provinces participating in the QUALICO-PC study [14]. Also, characteristics of participating family practitioners and practice settings were similar to those of all family practitioners in Quebec. The last data on practice profiles show 50% of family practitioners were female with a mean age of 50 years [24]. Generalizability of the results should be interpreted with caution; sampled family physicians may have worked in more highly performing practices than usual which could contribute to the overall high reported access. Information on the family practitioners and their practices that did not participate in the study was not available. Despite these limitations and overall low response rate, the data collected through the Canadian arm of QUALICO-PC represent the largest dataset on the quality and organization of primary care data in Canada. The patient sample was the same as the Quebec population for education and presence of a chronic illness. The female over-representation in the study is typical of recent cross-sectional studies in primary care in Canada [5, 25]. All the same, this gives us confidence in results of the study.

### Policy implications

Our findings suggest that supporting PHC organizations to increase same-day appointment and walk-in clinics could increase first-contact access for patients. This is consistent with the goals of the recent PHC reform, most notably the implementation of Family Medicine Groups [26]. With this implementation, different financial incentives for family practitioners were provided to increase their workload, to support practice in the primary care settings outside the hospitals, and to promote wide-spread use of advanced access scheduling [27].

## Conclusions

This study looked at what determines better first-contact accessibility at the organizational level in PHC organizations. First-contact accessibility increased most with the possibility for patients to have a same-day appointment or to walk-in the clinic. While patient-reported accessibility also increased with the number of hours worked per week by the family practitioner and the presence of physicians from other disciplines in the clinic, it was not associated with the use of electronic medical records or the presence of nurses. The advanced access model for practices is being implemented throughout Quebec and in the next years, it will be important to evaluate the effect of these changes on organizational characteristics and first-contact accessibility for patients.

## Abbreviations

PHC: Primary health care
QUALICO-PC: Quality and Costs of Primary Care
